# Treatment in the Case of Forcible Removal of the Uterus

**Published:** 1840-08

**Authors:** C. G. Ballard


### THE WESTERN JOURNAL.

No. VIII.

LOUISVILLE, AUGUST 1, 1840.

### DR. BALLARD'S CASE OF EXTIRPATED UTERUS.

We published in the June number of this Journal a brief notice of a remarkable case of forcible removal of the uterus by a midwife, which fell under the observation and treatment of Dr. Ballard, then of Westport, Kentucky, now of Indiana. Our notice has elicited from the Dr. a more circumstantial and authentic account of the case, which we take pleasure in laying before our readers, believing that it will be highly gratifying to them, as it was to us. The promptitude and vigour with which the inflammatory symptoms were met, and the rescue of the unfortunate sufferer from death after so serious a lesion, reflect great credit upon the skill of Dr. Ballard.

H. M.

Sir—I have just perused, in the June number of the Western Journal of Medicine and Surgery, a notice of a case of forcible removal of the uterus and its appendages after the expulsion of the fœtus, which occurred in Oldham county, Kentucky, communicated to you by my friend, Doctor Drane, which subsequently fell under my care. It was a case novel in its character, and worthy of being reported, and I intended to give it to the profession, and accordingly noted all the symptoms as they occurred at the time, with the minutiæ of medication, the character of the secretions, excretions, etc. But unfortunately my memorandum was destroyed by fire, some years since, and consequently I am unable, at this late period, to present the case to you in detail, as I am obliged to depend altogether upon memory.

This case so far as my reading extends is unique. It occurred about the 20th or 22d of June, 1828, in remarkably hot weather, the mercury ranging from 90° to 100° of Fah. The subject was thirty-two years of age and very corpulent. I saw her about 3 o'clock, P. M. Her breathing was short, difficult, and hurried; her pulse small, quick and thread-like; skin covered with perspiration; she complained of a sense of suffocation, but the hæmorrhage at the time was trifling. I gave her sixty drops of tincture opii which operated like a charm, her breathing becoming comparatively easy in a few minutes, and her pulse more slow, soft, and free from irritation. After she had rested for an hour or two upon the featherbed on which I found her, she was removed to one of straw, placed in a position to admit a free circulation of air.

About two hours after my arrival my patient became troubled with flatus in the stomach, of which she was relieved by a few grains of magnesia, and a little essence of peppermint. She slept some that night and appeared quite comfortable in the morning. The excessive perspiration had entirely subsided, but her skin was neither hot nor dry. I now directed an ounce of sulph. magnes. to be given every three hours until catharsis should be established, and cloths wet in cold water to be applied over the abdomen, so soon as the temperature of that region should rise above the natural standard. I then left her with a promise to return at 4 o'clock, P. M.

The salts I found had produced no effect, nor were there yet developed any symptoms of inflammation—no soreness, no increase of temperature—the patient free from pain and comfortable. She was ordered to continue the sulph. magnes.

It was now twenty-eight hours since the violence had been done, and I expected inflammation to supervene before morning, and in this I was not mistaken. About 12 o'clock I was summoned in haste; found my patient complaining of great pain and soreness in the abdomen, especially above the arch of the pubis; pulse full, strong and bounding; skin hot and dry. No action had been produced by the medicine upon the bowels. I immediately from a large orifice took thirty-six ounces of blood from the arm. Her pulse sunk, and she grew faint. An enema was administered which produced three or four copious discharges from the bowels, which, with the bleeding, relieved completely both the pain and arterial excitement. I directed the cold applications to be rigidly attended to, and gave the following powder every two hours: tart. antimon. $\frac{1}{4}$ gr., nit. potas. 8 grs., pulv. camph. 2 grs. The patient rested well the remainder of the night, and her pulse was quiet, and her skin cool and moist at 5 o'clock, A. M. At 6 the inflammatory action had returned with all its force, and I again took thirty-six ounces of blood from the arm, with the former decided and happy effects.

4 o'clock, P. M., the powders and cold applications have been continued; patient has taken a few spoonfuls of rice water; bowels have been moved three or four times; she had rested well until 3 o'clock, when slight arterial excitement came on, accompanied by a corresponding increase of pain and soreness in the abdomen, but the abstraction of twelve ounces of blood was sufficient to arrest it, and it returned no more. *Morning of the third day.*—Patient free from pain, pulse soft, skin moist, kidneys acting freely, tongue clean. She has had three or four copious dejections from the bowels during the night without any diminution of muscular strength. The antimonial to be continued.

The patient enjoyed from this time an uninterrupted convalescence, and although she continued to take the powders every two hours, for ten or twelve days, and had from six to ten discharges from the bowels every twenty-four hours, she experienced neither nausea nor prostration from their action. During this period the only nutriment she was permitted to take, was six or eight tablespoonfuls of rice water in the twenty-four hours. No secondary hœmorrhage occurred. When the sloughing stage came on, which was well marked by strong fœtor, followed by a moderate purulent discharge, an opposite treatment was observed; and under this she rapidly recovered, and has enjoyed good health, so far as I know, ever since. In this case, there was no lochial discharge, nor was lactation established; shewing the controlling influence of the uterus over this important secretion. Another interesting physiological fact.—Inquiries instituted two years after the recovery of my patient convinced me, that the accident had not entirely deprived her of sexual propensities: yet both the ovaria were removed with the uterus.

July, 1840. C. G. BALLARD.

